# The effect of health literacy on knowledge and receipt of colorectal cancer screening: a survey study

**DOI:** 10.1186/1471-2296-8-16

**Published:** 2007-03-30

**Authors:** David P Miller, Caroline D Brownlee, Thomas P McCoy, Michael P Pignone

**Affiliations:** 1 Department of Internal Medicine, Section of General Internal Medicine, Wake Forest University School of Medicine, Winston-Salem, North Carolina, USA; 2 Department of Medicine, Division of General Internal Medicine, Johns Hopkins University School of Medicine, Baltimore, Maryland, USA; 3 Department of Biostatistical Sciences, Section of Biostatistics, Wake Forest University School of Medicine, Winston-Salem, North Carolina, USA; 4 Department of Internal Medicine, Division of General Internal Medicine, University of North Carolina School of Medicine, Chapel Hill, North Carolina, USA

## Abstract

**Background:**

An estimated one-half of Americans have limited health literacy skills. Low literacy has been associated with less receipt of preventive services, but its impact on colorectal cancer (CRC) screening is unclear. We sought to determine whether low literacy affects patients' knowledge or receipt of CRC screening.

**Methods:**

Pilot survey study of patients aged 50 years and older at a large, university-affiliated internal medicine practice. We assessed patients' knowledge and receipt of CRC screening, basic sociodemographic information, and health literacy level. We defined limited literacy as reading below the ninth grade level as determined by the Rapid Estimate of Adult Literacy in Medicine. Bivariate analyses and exact logistic regression were used to determine the association of limited health literacy with knowledge and receipt of CRC screening.

**Results:**

We approached 105 patients to yield our target sample of 50 completing the survey (recruitment rate 48%). Most subjects were female (72%), African-American (58%), and had household incomes less than $25,000 (87%). Overall, 48% of patients had limited literacy skills (95% CI 35% to 61%). Limited literacy patients were less likely than adequate literacy patients to be able to name or describe any CRC screening test (50% vs. 96%, p < 0.01). In the multivariable model, limited literacy patients were 44% less likely to be knowledgeable of CRC screening (RR 0.56, p < 0.01). Self-reported screening rates were similar (54% vs. 58%, p = 0.88).

**Conclusion:**

Patients with limited literacy skills are less likely to be knowledgeable of CRC screening compared to adequate literacy patients. Primary care providers should ensure patients' understanding of CRC screening when discussing screening options. Further research is needed to determine if educating low literacy patients about CRC screening can increase screening rates.

## Background

Routine screening for colorectal cancer (CRC) can decrease mortality and is widely advocated [1,2]. However, only half of Americans receive the recommended screening for this preventable disease[3,4]. For patients with low health literacy, defined as the ability to read and interpret information needed to make healthcare decisions, CRC screening rates may be even lower[5].

Over one third of adults in the United States have inadequate literacy skills [5-9]. Prior studies have found that low literacy female patients are less likely to understand commonly recommended cancer screening tests such as Papanicolaou smears and mammograms[10,11]. Others have reported that low literacy patients are less likely to receive commonly recommended preventive care interventions[12,13].

Less is known about the association of health literacy with knowledge and receipt of CRC screening. Two quantitative studies examined the association of health literacy with knowledge of CRC screening and reported mixed results[14,15]. Only one of these studies examined actual screening rates and found that patients with inadequate health literacy were less likely to have ever been screened for CRC[15]. However, after adjusting for other sociodemographic characteristics, the effect of literacy was not significant.

Additional studies are needed to determine if CRC screening interventions targeting low literacy populations are warranted. To prepare for a subsequent large trial, we conducted a pilot survey study to further explore the association of health literacy with CRC screening knowledge and behavior.

## Methods

### Participants

We conducted the study in September 2004 at a large university-affiliated community-based internal medicine faculty-resident practice. All English-speaking patients aged 50 years or older were eligible to participate. After patients were placed in an exam room, a research assistant attempted to approach all eligible patients to invite them to participate. Patients with obvious cognitive or physical impairments that would interfere with their ability to complete the survey were excluded. All participants were given a five dollar gift card to a national discount store. The Wake Forest University Institutional Review Board approved the study protocol, and all participants gave verbal informed consent.

### Measurements

We created a survey instrument containing 26 items that assessed patients' knowledge of CRC screening interventions, their prior experiences with screening, their methods for learning about health topics (brochures, nurse counseling, physician counseling, video programs, and internet or computer programs), and basic sociodemographic information including health literacy level. Literacy was measured using the Rapid Estimate of Adult Literacy in Medicine (REALM), a previously validated instrument that includes a list of 66 health terms and requires approximately two to three minutes to complete [16]. We defined limited literacy as reading below the ninth grade level. Patients who declined to participate had only their age, gender, and race recorded. Specific reasons for refusing to participate were not elicited.

Basic knowledge of CRC screening was assessed by two questions: "Did you know that doctors can test people to see if they have colon (or large bowel) cancer?" and "Can you tell me the name of a test (or how the test is performed) that doctors use to look for colon cancer?" Acceptable tests included fecal occult blood testing (FOBT), flexible sigmoidoscopy, and colonoscopy. We excluded barium enema from the survey due to the study clinic's infrequent use of this screening test. Participants were coded as knowledgeable if they could name or describe in simple terms any of the screening tests. The surveyor then described the relevant screening tests and asked the participants when they last received them, if ever. Completed screening was defined as receiving FOBT within the last year, flexible sigmoidoscopy within five years, or colonoscopy within 10 years.

A research assistant or member of the research team administered each survey in a private setting. Literacy and demographic data were collected at the completion of the survey to keep the surveyor blinded to literacy level. All survey data was double-entered into a database and cross-checked for accuracy.

### Statistical Analyses

Because we conducted this study to prepare for a subsequent larger trial, our primary outcomes of interest were the percentage of patients with limited health literacy, the percentage of patients who could name or describe any relevant CRC screening test, and the percentage who had received screening. Based on prior published studies in similar populations, we assumed the prevalence of limited health literacy would be approximately 50% [6,7]. To estimate the actual prevalence to within +/- 15%, we calculated a target sample size of 50 patients.

Descriptive and bivariate analyses were performed for the outcomes above using chi-square and Fisher's Exact tests. Confidence intervals for proportions were calculated using Wilson-Score methods[17]. We used exact logistic regression to determine the association of limited health literacy with knowledge and receipt of CRC screening. To construct the logistic regression model, we first examined the bivariate association of literacy level, knowledge, and receipt of CRC screening with each possible covariate (age, gender, race, marital status, health insurance, household income, provider type, and frequency of visits). Variables significant at the 5% level from the bivariate analyses were included in the final multivariable logistic regression model. Given that education is highly correlated with literacy, we did not include education in the multivariable model to avoid over-controlling for this predictor. Exact logistic regression was performed using the network method described by Mehta et al[18]. Estimates of adjusted relative risk for knowledge and receipt of CRC screening were obtained using Cochran-Mantel-Haenszel methods since multivariable modeling resulted in at most only one other covariate additional to literacy level. All analyses were performed using SAS version 9.1.3 (SAS Institute, Cary, NC, USA).

## Results

During 12 clinic sessions, we approached 105 subjects to reach our target of 50 completed surveys (48% response rate). Non-participants were similar to participants by age (mean age 63.3 years versus 62.5 years; p = 0.67). Most non-participants and participants were female (58% versus 72%, p = 0.14) and African-American (75% versus 58%, p = 0.07).

Nearly all participants reported household incomes of less than $25,000 (87%). Limited literacy was present in 48% of participants (95% CI 35% to 61%); 26% (13/50) read below the 7^th ^grade level, and an additional 22% (11/50) read at the 7^th ^to 8^th ^grade level.

Table 1 displays patients' characteristics by literacy level. Overall, limited literacy patients were more likely to be African-American and less educated. Differences in marital status approached but did not reach significance.

**Table 1:** Patient characteristics by literacy level

**Patient Characteristic**	**Limited Literacy** **N (%) (n = 24)**	**Adequate Literacy** **N (%) (n = 26)**	p-value*
Age, years. Mean (SD)	62.9 (10.5)	62.2 (9.2)	0.78

Gender			0.86
Female	17 (71)	19 (73)	
Male	7 (29)	7 (27)	

Race			0.02
African-American	18 (75)	11 (42)	
Caucasian	6 (25)	15 (58)	

Marital Status			0.12
Married/living together	3 (13)	8 (31)	
Not married	21 (88)	18 (69)	

Education Level			<0.01
< 12 years education	17 (71)	8 (31)	
High school graduate	7 (29)	6 (23)	
> High school graduate	0 (0)	12 (46)	

Insurance Status^†^			0.49
Uninsured	6 (25)	4 (15)	
Medicare	11 (46)	14 (54)	
Medicaid	9 (38)	14 (54)	
Commercial/Military	5 (21)	6 (23)	

Household Income			0.67
< $25,000	19 (79)	21 (81)	
$25,000 or more	2 (8)	4 (15)	

Provider Type			0.88
Physician assistant	6 (25)	7 (27)	
Resident physician	8 (33)	7 (27)	
Attending physician	10 (42)	12 (46)	

Frequency of medical visits			0.29
< 4 visits/year	8 (33)	5 (20)	
4 or more visits/year	16 (67)	20 (80)	

CRC screening current ^‡^			0.80
Yes	13 (54)	15 (58)	
No	11 (46)	11 (42)	

*A t-test for difference in means was used to compare age; all other variables were compared using Chi-Square and Fisher's Exact tests.

^† ^Because some patients have more than one type of insurance, percentages add to greater than 100%; p-value is for test of any insurance vs. no insurance

^‡ ^Current screening defined as receiving fecal occult blood testing within the last year, flexible sigmoidoscopy within the last five years, or colonoscopy within the last 10 years.

Compared to adequate literacy patients, patients with limited literacy skills were much less likely to be able to name any CRC screening test (13% versus 69%, p < 0.01) or describe any CRC screening test (50% versus 96%, p < 0.01). The only other characteristics associated with knowledge of CRC screening in bivariate analyses were race and education (Table 2). After controlling for race in logistic regression models, only literacy level was significantly associated with knowledge of CRC screening. Limited literacy patients were 79% less likely to be able to name any screening test, and 44% less likely to be able to describe any screening test (Table 3).

**Table 2:** Bivariate analysis of patient characteristics by knowledge of CRC tests*

	**Able to Name a CRC Test**			**Able to Describe a CRC test**		
	
**Patient Characteristic**	**Yes N (%)** **(n = 21)**	**No N (%)** **(n = 29)**	**p-value^†^**	**Yes N (%)** **(n = 37)**	**No N (%)** **(n = 13)**	**p-value^†^**
Age, years. Mean (SD)	60.9 (9.9)	63.7 (9.6)	0.31	63.2 (10.1)	60.5 (8.8)	0.40

Gender			0.57			1.00
Female	16 (76)	20 (69)		27 (73)	9 (69)	
Male	5 (24)	9 (31)		10 (27)	4 (31)	

Race			0.02			0.02
African-American	8 (38)	21 (72)		18 (49)	11 (85)	
Caucasian	13 (62)	8 (28)		19 (51)	2 (15)	

Marital Status			0.10			1.00
Married/living together	7 (33)	4 (14)		29 (78)	10 (77)	
Not married	14 (67)	25 (86)		8 (22)	3 (23)	

Health Literacy			<0.01			<0.01
Limited (<9^th ^grade)	3 (14)	21 (72)		12 (32)	12 (92)	
Adequate (> = 9^th ^grade)	18 (86)	8 (28)		25 (68)	1 (8)	

Education Level			0.01			0.09
< 12 years	6 (29)	19 (66)		15 (41)	10 (77)	
High school graduate	6 (29)	7 (24)		11 (30)	2 (15)	
> High school graduate	9 (43)	3 (10)		11 (30)	1 (8)	

Insurance Status^‡^			1.00			0.71
Uninsured	4 (19)	6 (21)		7 (19)	3 (23)	
Medicare	12 (57)	13 (45)		20 (54)	5 (38)	
Medicaid	11 (52)	12 (41)		17 (46)	6 (46)	
Commercial/Military	3 (14)	7 (24)		7 (19)	3 (23)	

Household Income			1.00			1.00
< $25,000	17 (81)	23 (79)		30 (81)	10 (77)	
$25,000 or more	3 (14)	3 (10)		5 (14)	1 (8)	

Provider Type			0.07			0.08
Physician assistant	2 (10)	11 (38)		10 (27)	3 (23)	
Resident physician	7 (33)	8 (28)		8 (22)	7 (54)	
Attending physician	12 (57)	10 (34)		19 (51)	3 (23)	

Frequency of visits			0.71			0.29
< 4 visits/year	5 (24)	8 (28)		8 (22)	5 (38)	
4 or more visits/year	16 (76)	20 (69)		28 (76)	8 (62)	

* Relevant screening tests include fecal occult blood testing, flexible sigmoidoscopy, and colonoscopy.

^† ^A t-test for difference in means was used to compare age; all other variables were compared using Chi-Square and Fisher's Exact tests.

^‡ ^Because some patients have more than one type of insurance, percentages add to greater than 100%.

**Table 3:** Knowledge and receipt of CRC screening by literacy level

**Outcome**	**Limited Literacy** **N (%) (n = 24)**	**Adequate Literacy** **N (%) (n = 26)**	RR (95% CI)*	**p-value†**
Able to name a CRC screening test^‡^	3 (13)	18 (69)	0.21 (0.07, 0.64)	<0.01
Able to name or describe CRC screening test	12 (50)	25 (96)	0.56 (0.38, 0.83)	<0.01
Currently screened^£^	13 (54)	15 (58)	0.99 (0.64, 1.55)	0.88

*Adjusted Relative Risk using Cochran-Mantel-Haenszel methods: RR adjusted for race for knowledge outcomes; and age group (median split; median age = 61.5 yrs.) for receipt of screening.

†p-value from Cochran-Mantel-Haenszel test

‡Relevant screening tests include fecal occult blood testing (or stool test kit), flexible sigmoidoscopy, and colonoscopy.

£ Currently screened defined as receiving fecal occult blood testing within the last year, flexible sigmoidoscopy within the last five years, or colonoscopy within the last 10 years.

In both the bivariate and multivariate analyses, there was no significant difference in self-reported receipt of screening between limited literacy and high literacy patients (54% vs. 58%, 95% Wilson CI for difference -29% to +22%). In bivariate analyses, only age was associated with receipt of screening (mean age for screened patients 66.5 years versus 57.4 years, p < 0.01). The vast majority (83%) of patients who received CRC screening reported that their physician suggested it. Only five of the 36 screened patients reported asking their physicians for the test (data not shown).

## Discussion

In summary, we found that patients with limited health literacy were approximately 50% less likely than adequate literacy patients to be knowledgeable of any CRC screening test. However, we detected no significant difference in self-reported screening rates.

Our finding that limited literacy patients had decreased knowledge of CRC screening is consistent with the results of the few published studies in this area. In separate reports, Lindau and Davis found that low literacy women had less knowledge of cervical cancer screening and mammography [10,11]. Similarly, Dolan and Guerra reported that low literacy men were less likely to know about CRC screening tests [14,15]. However, in Guerra's report, the association of literacy with CRC screening knowledge became non-significant after adjusting for other sociodemographic characteristics [15]. In our analysis, literacy remained a significant predictor of knowledge even after controlling for other patient factors. One possible explanation for this difference is that Guerra controlled for education whereas we did not due to the high collinearity between education and literacy.

Even though low literacy patients in our study were much less knowledgeable of CRC screening, self-reported screening rates were similar. It is possible that subjects over-reported the screening they received, as has been found by others [19]. Any over-reporting would decrease our ability to detect a difference in screening rates. Our study also may have been hampered by a lack of power. However, CRC screening rates differed by only 4% in our study, and our confidence interval suggests that we were unlikely to have missed any true difference of 30% or greater.

Alternatively, the similarity in screening rates we observed may reflect physician recommendation. Prior studies have reported that a physician's recommendation is a powerful predictor of patients receiving screening [14,15]. Similarly, in our study, over 80% of patients who received CRC screening reported that their physician suggested it. Because patients rarely reported requesting screening themselves, educational interventions designed to activate patients may help increase screening rates. Current screening appears to be dependent on physicians' recommendations.

Our study does have additional limitations. First, because this is a pilot study, our sample size was relatively small and may not be representative of other populations. Despite the smaller sample size, our estimated prevalence of limited literacy is consistent with larger studies[9], and our results for knowledge are highly significant. Second, our study may be subject to selection bias if low literacy patients or those less knowledgeable about health topics were reluctant to participate. This type of selection bias would cause us to overestimate the prevalence of adequate literacy and patient's knowledge. Third, administered surveys can be subject to interviewer bias. To minimize this risk, we did not assess literacy level or demographic factors such as educational attainment until the end of each survey. Furthermore, our measured outcome of ability to name or describe any CRC screening test required little subjective interpretation. Lastly, for this pilot study, we focused on patients' knowledge of CRC screening and not their attitudes and beliefs, which could also influence their motivation to receive screening or learn about screening options.

## Conclusion

In conclusion, we found that patients with limited health literacy were much less likely to be knowledgeable of any CRC screening test compared to adequate literacy patients. Because patients rarely reported requesting CRC screening, current screening rates appear dependent on health care providers' recommendations. Further research is needed to determine if educating low literacy patients about CRC screening can increase screening rates. In addition, given the high prevalence of limited literacy, primary care providers should ensure patients' understanding of CRC screening when discussing screening options.

## Competing interests

The author(s) declare that they have no competing interests.

## Authors' contributions

DM conceived of the study and its design, oversaw the analysis and interpretation of the data, and drafted the manuscript. CB contributed to the study design, assisted with data collection and interpretation, and helped draft the manuscript. TM performed the statistical analysis, contributed to data interpretation, and helped draft the manuscript. MP contributed to the study design, data interpretation, and drafting of the manuscript. All authors read and approved the final manuscript.

## Pre-publication history

The pre-publication history for this paper can be accessed here:


